# Deciphering the Role of SLFN12: A Novel Biomarker for Predicting Immunotherapy Outcomes in Glioma Patients Through Artificial Intelligence

**DOI:** 10.1111/jcmm.70317

**Published:** 2024-12-30

**Authors:** Zigui Chen, Chao Liu, Wei Zheng, Yuhua Fang, He Zhang, Jiawei Luo, Jiale Li, Yingqi Qiu, Jun Peng, Ying Xia, Changfeng Miao, Qisheng Luo

**Affiliations:** ^1^ Department of Neurosurgery Haikou Affiliated Hospital of Central South University Xiangya School of Medicine Haikou China; ^2^ Department of Neurosurgery Central Hospital of Zhuzhou Zhuzhou Hunan China; ^3^ Department of Neurosurgery Affiliated Hospital of Youjiang Medical University for Nationalities Baise Guangxi China; ^4^ Guangxi Engineering Research Center for Biomaterials in Bone and Joint Degenerative Diseases Baise Guangxi China; ^5^ Department of Clinical Research Center Haikou Affiliated Hospital of Central South University Xiangya School of Medicine Haikou China; ^6^ Department of Neurosurgery Second Branche Hunan Provincial People's Hospital (The First Affiliated Hospital of Hunan Normal University) Changsha Hunan China

**Keywords:** artificial intelligence, gliomas, immunotherapy, SLFN12, tumour microenvironment

## Abstract

Gliomas are the most prevalent form of primary brain tumours. Recently, targeting the PD‐1 pathway with immunotherapies has shown promise as a novel glioma treatment. However, not all patients experience long‐lasting benefits, underscoring the necessity to discover reliable biomarkers for predicting treatment outcomes. This study applied a range of advanced artificial intelligence methods to identify a new biomarker linked to the effectiveness of anti‐PD‐1 immunotherapy in glioma patients. Through an extensive analysis of single‐cell RNA sequencing and bulk transcriptomic data from over 3000 patients, the gene SLFN12 emerged as a significant and independent predictor of immunotherapy response. Our results indicate that elevated SLFN12 expression is associated with worse overall survival across various glioma cohorts. Notably, we found that patients with high SLFN12 levels are less likely to respond favourably to anti‐PD‐1 treatment, positioning SLFN12 as a clinically valuable biomarker for personalised treatment decisions. Functional studies revealed that SLFN12 is involved in key immune‐related pathways, shedding light on its potential role in altering the tumour microenvironment and impacting immunotherapy outcomes. Additional laboratory experiments confirmed the role of SLFN12 in promoting glioma cell proliferation, migration and macrophage recruitment. In summary, this study identifies SLFN12 as a novel biomarker for predicting immunotherapy response in glioma patients, offering new insights for precision immunotherapy approaches.

## 1 Introduction

Gliomas represent the most prevalent category of primary brain tumours, with high‐grade variants like glioblastoma (GBM) being some of the most aggressive and deadly cancers [1]. Despite advances in standard‐of‐care treatment, typically involving surgical resection, radiation and chemotherapy, the median overall survival for patients with GBM remains only 12–15 months [2, 3].

Recently, immunotherapies targeting the programmed cell death‐1 (PD‐1) pathway have emerged as a promising new treatment approach for gliomas [4]. However, the clinical benefit of anti‐PD‐1 therapies has been modest, with only a subset of patients exhibiting durable responses [5]. Understanding the biological factors determining a patient's response to immunotherapy is crucial for improving patient selection and therapeutic outcomes [6].

Several studies have investigated potential immunotherapy response biomarkers in gliomas, including tumour mutational burden, expression of PD‐L1 and immune cell infiltration [7, 8, 9]. However, the complex and heterogeneous nature of the tumour microenvironment in gliomas has made it challenging to identify robust, predictive biomarkers using conventional approaches. Machine learning techniques offer a promising avenue to address this challenge, as they can uncover intricate patterns in high‐dimensional data that may not be discernible through traditional statistical analyses [10]. In other words, machine learning techniques facilitate the analysis of large genomic datasets, identifying mutations and biomarkers that are crucial for understanding cancer at the molecular level.

This study employed a series of artificial intelligence‐related methods, applied to over 3000 patients, to identify a novel biomarker that predicts response to anti‐PD‐1 immunotherapy in glioma patients. Our findings demonstrate that the level of the Schlafen Family Member 12 (SLFN12) is a strong and independent predictor of immunotherapy response in gliomas. These results have important implications for patient selection and the development of precision immuno‐oncology strategies for this devastating disease.

## 2 Methods

### 2.1 Data Collection and Procession

The Smart‐seq2 software was employed to analyse the single‐cell RNA sequencing (scRNA‐seq) data from human glioblastoma (GBM) samples sourced from the Single Cell Portal (SCP50 and SCP393). Additionally, The Cancer Genome Atlas (TCGA), Chinese Glioma Genome Atlas (CGGA) and Gene Expression Omnibus (GEO) databases retrieved bulk sequencing data for human glioma samples.

### 2.2 Computational Analysis

The raw data of the GBM immune checkpoint blockade (ICB) cohort (PRJNA482620) was transformed into Transcripts Per Million (TPM) format [11]. ICB‐associated genes between responders and non‐responders in PRJNA482620 were identified using the R package limma [12]. The Single‐Sample Gene Set Enrichment Analysis (ssGSEA) was performed to calculate the ICB score based on the ICB‐associated genes. Weighted Gene Co‐expression Network Analysis (WGCNA) was performed to determine the ICB‐related genes [13]. Soft threshold settings were implemented to guarantee a network topology free of scaling and producing a TOM matrix. The parameter was set to a power of *β* = 10. Genes in the blue module were taken out for further exploration. Eight immune indexes were collected, including CYT [14], IFNγIS [15], AyersExpIS [15], T cell–inflamed signature [15], RohIS [16], DavoliIS [17], RIR [18] and ImmuneScore [19]. Genes in the blue module that were positively correlated with all eight immune markers were further analysed as ICB‐related genes. Then after Unicox analysis, prognosis‐related ICB‐related genes are filtered out. For further downscaling, three prognosis machine learning methods, Cox Proportional Hazards Boosting (CoxBoost), Random Survival Forest (RSF) [20] and Least Absolute Shrinkage and Selection Operator (LASSO) [21] were applied. CoxBoost is a boosting algorithm specifically designed for survival analysis. It enhances the prediction of survival outcomes based on covariates. RSF is an ensemble learning method that extends random forests to handle time‐to‐event data. It accounts for censoring and provides variable importance measures. LASSO is used for regression and variable selection, helping to improve model interpretability by regularising the coefficients. The genes recognised by all three machine learning methods were screened out as candidate genes for subsequent analysis. Metascape was used to perform functional annotation of SLFN12 [22]. Gene Set Enrichment Analysis (GSEA) was applied to SLFN12. This widely used computational method determines whether a predefined set of genes shows statistically significant differences in expression under two biological conditions. The R package oncoPredict predicted chemotherapy drug responses related to SLFN12 [23]. GISTIC 2.0 analysis was performed [24]. The R package maftools generated the mutation landscape related to SLFN12 [25]. The immune cells by TIMER, MCPcounter and ssGSEA were calculated independently [26, 27, 28]. The Submap (Subnetwork Mappings in Alignment of Pathways) was used to determine if SLFN12 can affect PD‐1 and CTLA‐4 ICB therapies [29]. The related annotation data were obtained from the previous research [30]. The R package Seurat generated the Uniform Manifold Approximation and Projection (UMAP) and Vlnplot in the scRNA‐seq data [31].

### 2.3 In Vitro Validation on SLFN12

The HMC3 microglial cell line and the glioma cell lines U251 and LN229 were obtained from iCell. SLFN12 was silenced using two siRNA sequences (Forward GAACAGAACUUGAUCGGAATT; Forward GGAGAGAACAGUAGGAAAATT). Total RNA was extracted from glioma cells treated with siRNA. This RNA was then reverse‐transcribed into cDNA using a reverse transcriptase enzyme. The resulting cDNA served as a quantitative PCR (qPCR) amplification template. The abundance of target gene transcripts was assessed using gene‐specific primers. Real‐time monitoring during the qPCR process allowed for precise quantification of mRNA levels. Relative expression was calculated using the 2^−ΔΔCt^ method, normalising against endogenous control genes.

The EdU assay was performed to evaluate cell division rates. Glioma cells were treated with EdU, a thymidine analogue that incorporates into the DNA of cells in the S‐phase. After staining, cells were observed under a microscope. The percentage of EdU‐positive cells provided a quantitative measure of the proliferating cell population.

The migratory capacity of glioma cells was assessed using a Transwell assay. Cells were placed in the upper chamber of a Transwell plate with a permeable membrane, and those that migrated to the lower compartment were quantified. Additionally, a co‐culture Transwell assay was utilised to examine the migration potential of macrophages. In this setup, macrophages were seeded in the upper chamber, while glioma cells occupied the lower chamber, with migration assessed similarly.

### 2.4 Statistical Analysis

With R, all statistical analyses were carried out. The normally distributed variables and non‐normally distributed data across the two groups were compared using the Student's *t*‐test and Wilcoxon test, respectively. Statistics were deemed significant when *p* < 0.05.

## 3 Results

### 3.1 WGCNA for ICB‐Related Genes

**FIGURE 1:**
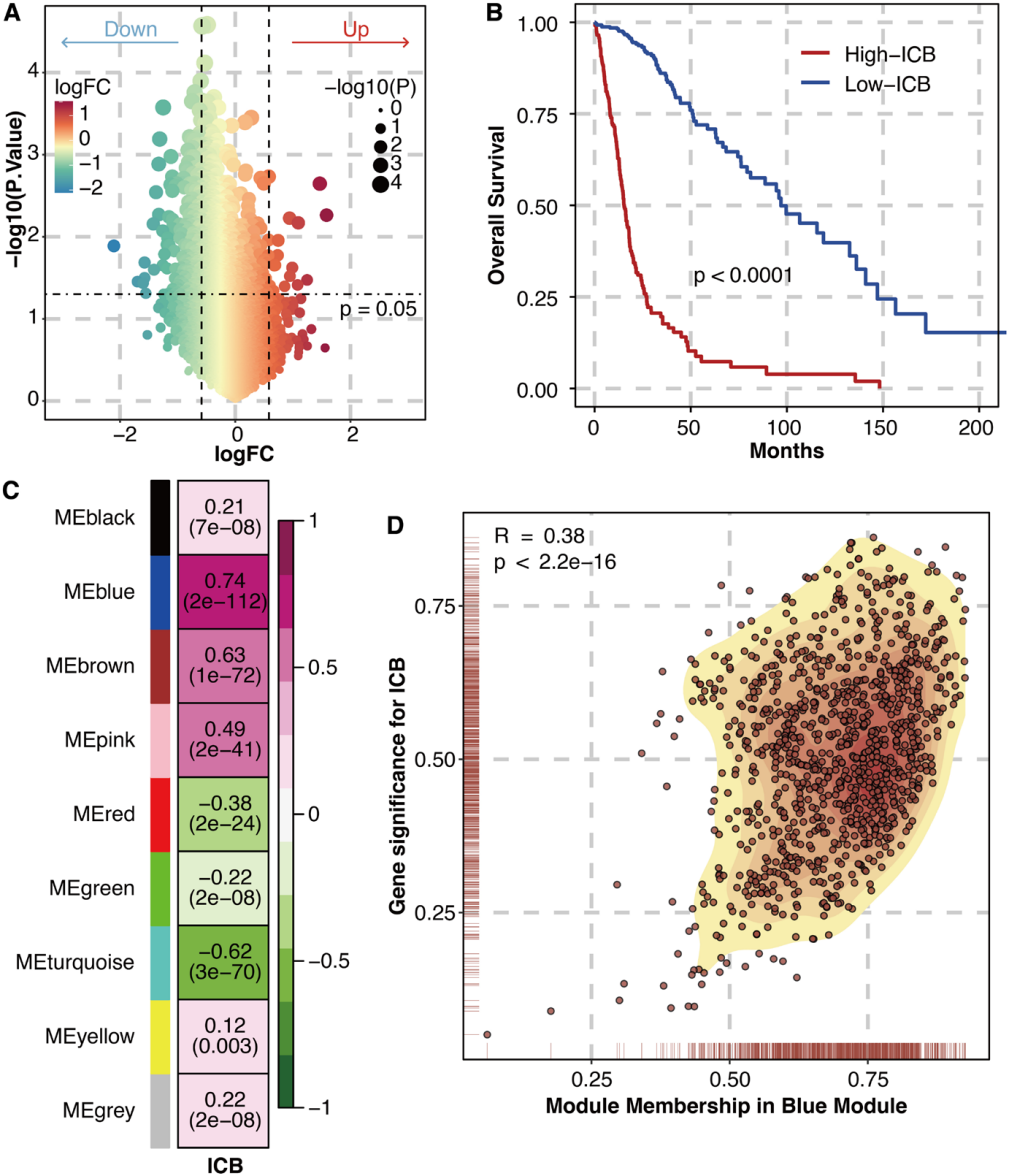
WGCNA for ICB‐related genes. (A) DEGs between the ICB response group and the ICB non‐response group. (B) Survival curves of high and low ICB score groups in the TCGA glioma dataset. (C) Module‐ICB relationship. (D) Correlation between module membership and gene significance in the blue module.

DEGs between the ICB response group and the ICB non‐response group were identified in the GBM immunotherapy cohort PRJNA482620 (Figure 1A). The ICB score was calculated based on the ICB‐associated genes using ssGSEA. The high ICB score group had a significantly shortened survival time in the TCGA glioma dataset (Figure 1B). The module‐ICB relationship revealed that the ICB score was most associated with the blue module (Figure 1C). The module membership was significantly associated with gene significance in the blue module (Figure 1D).

### 3.2 Machine Learning for Potent Genes

**FIGURE 2:**
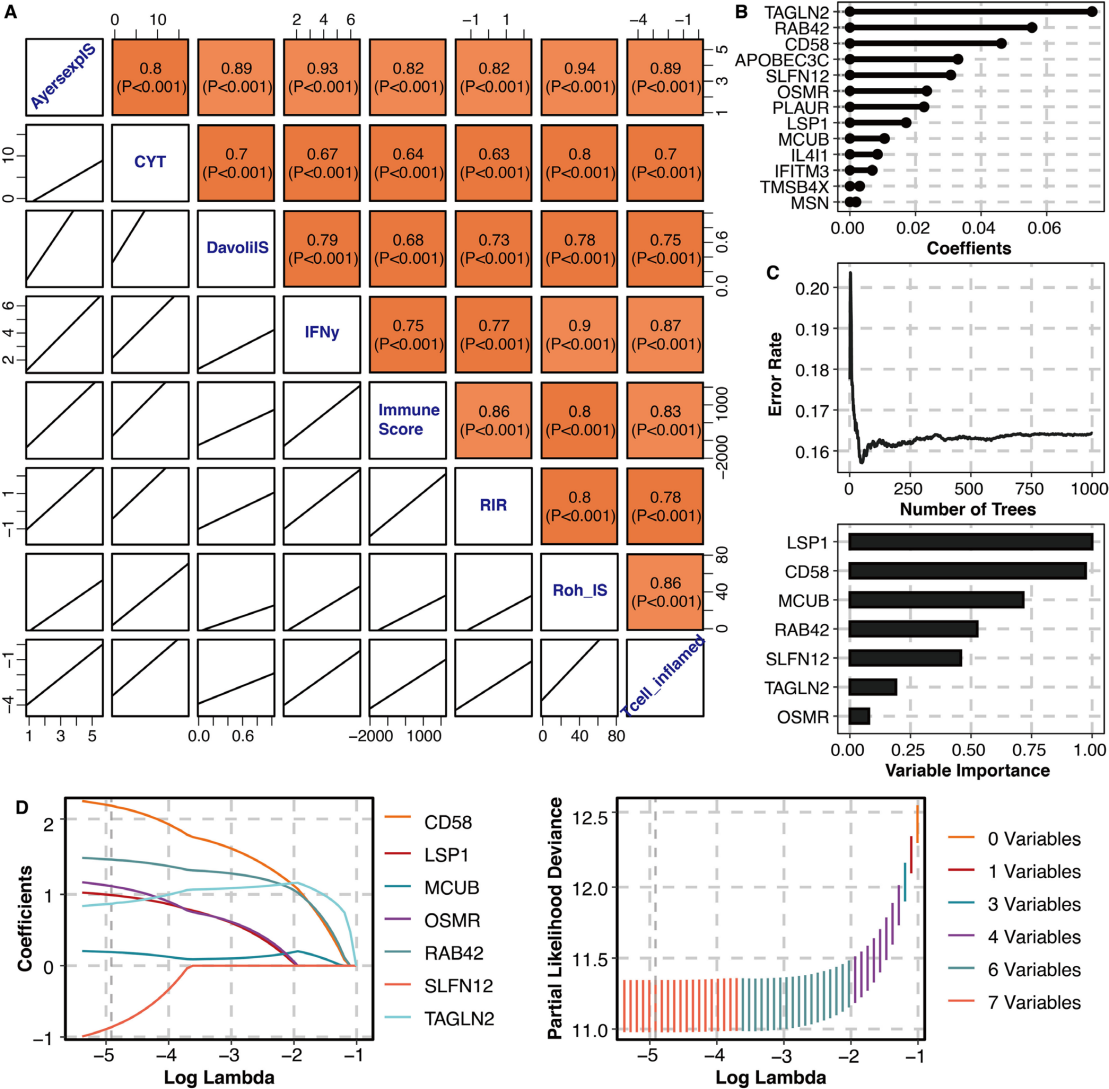
Machine learning for potent genes. (A) Correlation between blue module genes and eight immune indexes. (B) CoxBoost analysis on ICB‐related genes. (C) Random forest analysis on ICB‐related genes. (D) LASSO regression analysis on ICB‐related genes.

Correlation between blue module genes and eight immune indexes is shown in Figure 2A, in which module genes positively correlated with all eight immune indexes, including CYT, IFNγIS, AyersExpIS, T cell–inflamed signature, RohIS, DavoliIS, RIR and ImmuneScore, were extracted as ICB‐related genes for further analysis. Then after Unicox analysis, prognosis‐related ICB‐related genes are filtered out. CoxBoost analysis was performed on prognosis‐related ICB‐related genes for dimension reduction, resulting in 13 potent genes (Figure 2B). RSF analysis was performed on prognosis‐related ICB‐related genes, including seven potent genes (Figure 2C). LASSO regression analysis was performed on prognosis‐related ICB‐related genes, finally reaching seven potent genes (Figure 2D).

### 3.3 Prognostic Value of SLFN12

**FIGURE 3:**
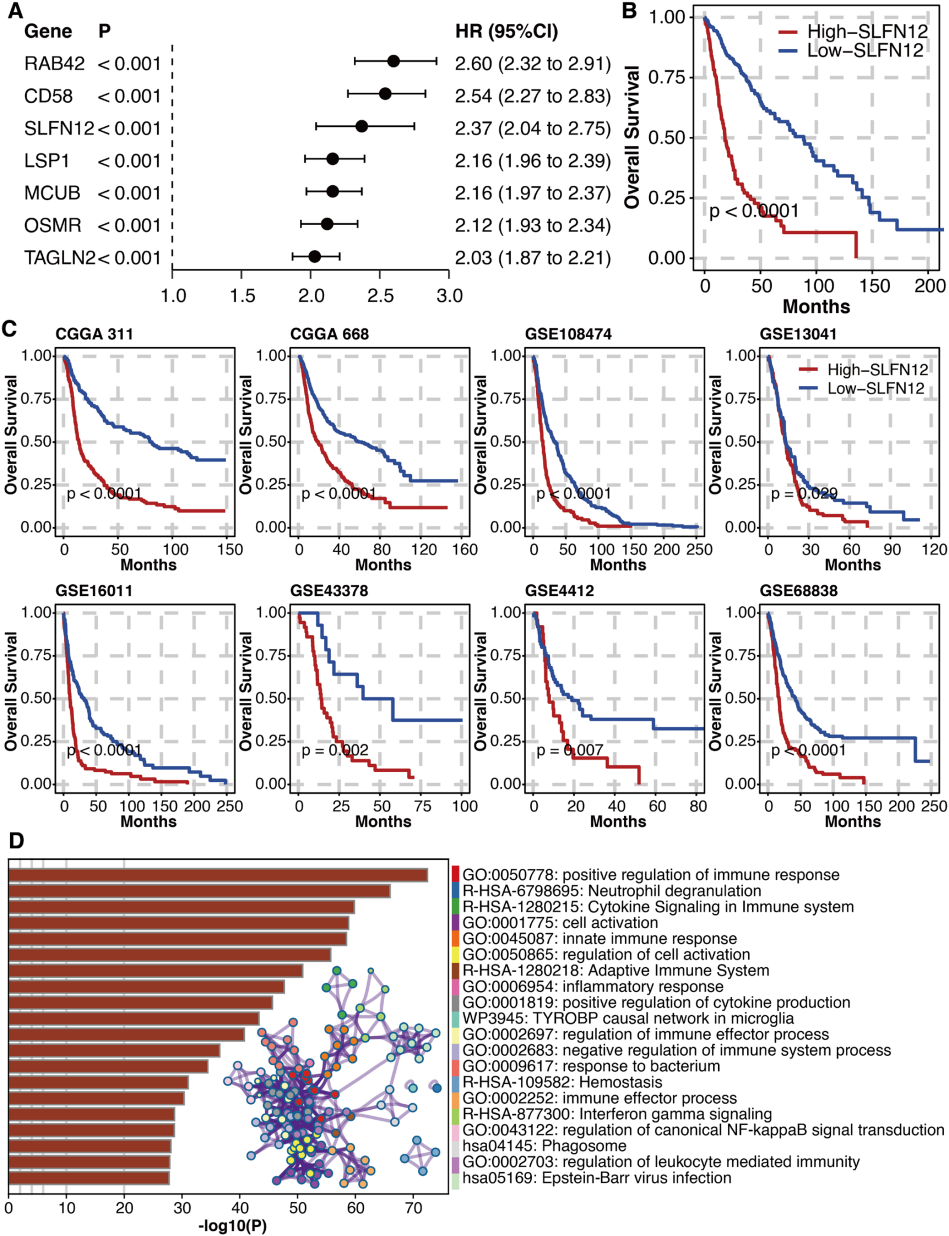
Prognostic value of SLFN12. (A) Univariate Cox regression analysis on potent genes. (B) Survival curves of high and low SLFN12 groups in the TCGA glioma dataset. (C) Survival curves of high and low SLFN12 groups in different glioma datasets. (D) metascape‐based functional annotation of SLFN12.

**FIGURE 4:**
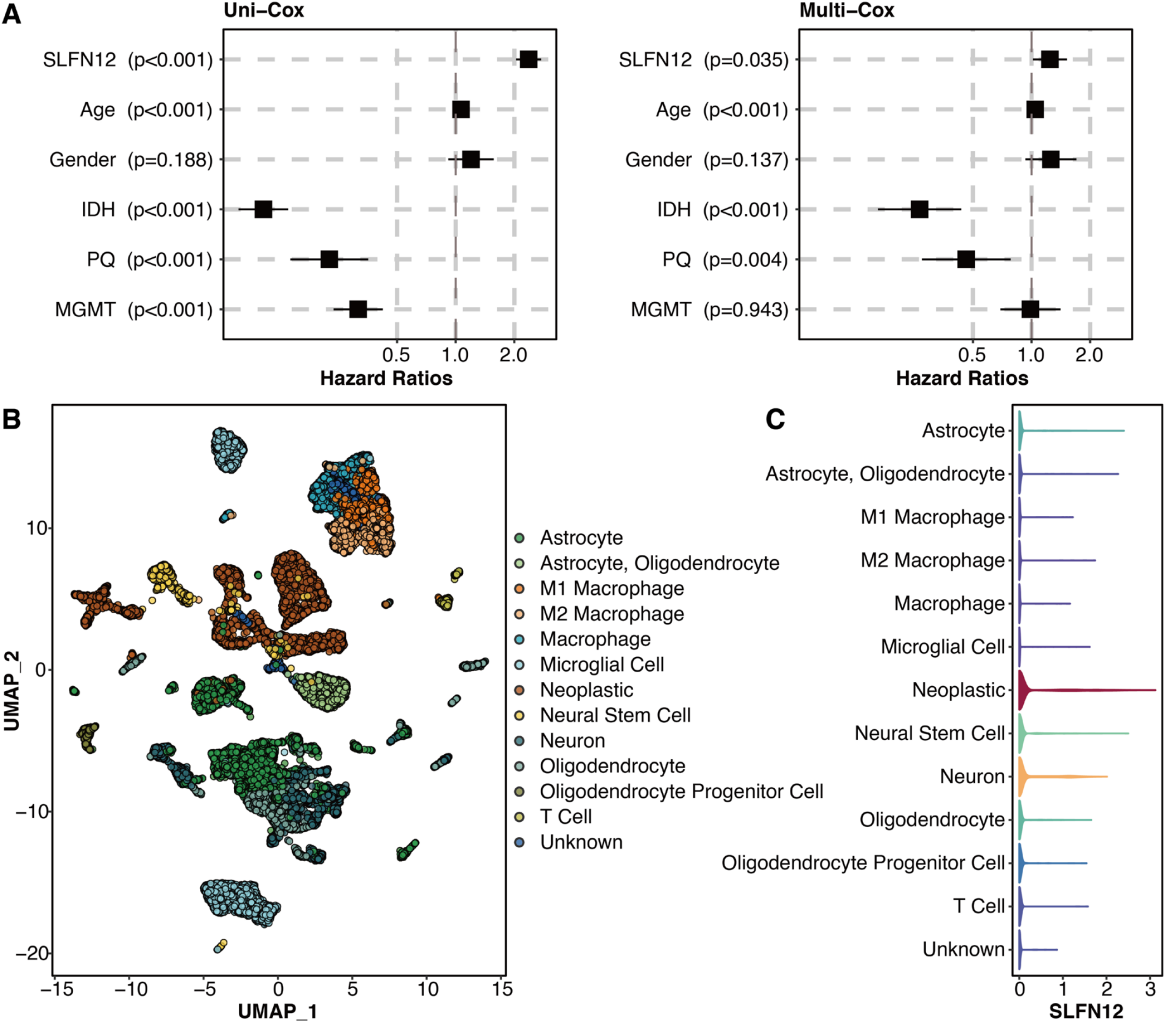
scRNA‐seq analysis on SLFN12. (A) Univariate and multivariate Cox regression analysis on SLFN12 and clinical factors. (B) UMAP shows the microenvironment cells in the GBM scRNA‐seq dataset. (C) Vlnplot shows the SLFN12 expression in the microenvironment cells in the GBM scRNA‐seq dataset.

Univariate Cox regression analysis was performed on potent genes, of which all seven genes were hazardous (Figure 3A). The high SLFN12 group had a significantly shortened survival time in the TCGA glioma dataset (Figure 3B). The high SLFN12 group had significantly shortened survival times in different glioma datasets (Figure 3C). The metascape‐based functional annotation of SLFN12 revealed that SLFN12 was associated with immune activity (Figure 3D). GSEA on SLFN12 was conducted, and important pathways such as immunological response, T cell receptor signalling route, B cell receptor signalling pathway, dendritic cell chemotaxis and response to cytokine were considerably enriched (Figure S1). Univariate and multivariate Cox regression analyses were performed on SLFN12 and clinical factors, of which SLFN12 and age were hazardous (Figure 4A).

### 3.4 The scRNA‐Seq Analysis on SLFN12

UMAP shows the microenvironment cells in the GBM scRNA‐seq dataset (Figure 4B). Vlnplot shows the SLFN12 expression in the microenvironment cells in the GBM scRNA‐seq dataset, in which SLFN12 was highly expressed in tumour cells (Figure 4C).

### 3.5 In Vitro Validation on SLFN12

**FIGURE 5:**
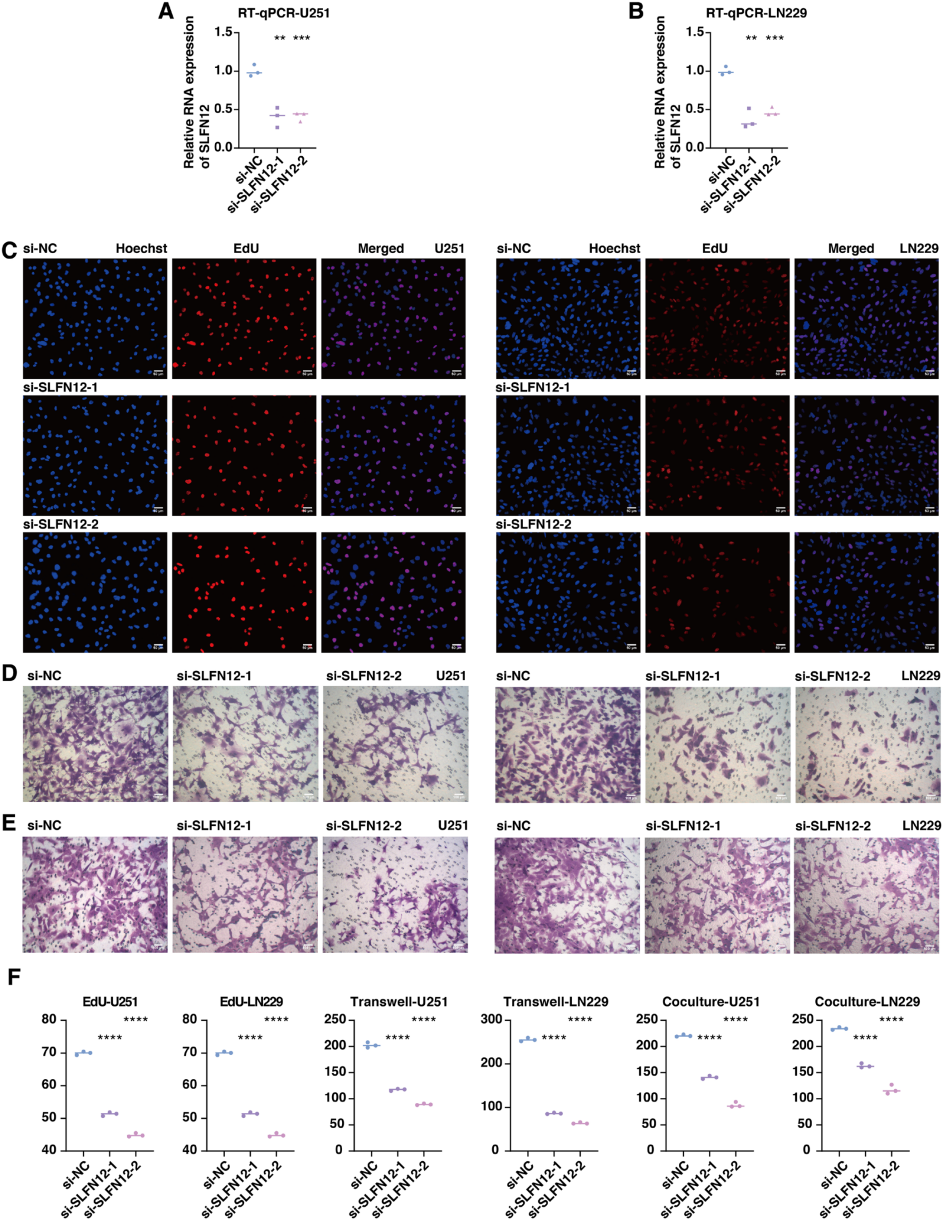
In vitro validation on SLFN12. (A) RT‐qPCR assay shows the RNA expression of SLFN12 in different groups in U251 cells. (B) RT‐qPCR assay shows the RNA expression of SLFN12 in different groups in LN229 cells. (C) EdU assay shows the proliferated glioma cells in different groups in U251 and LN229 cells. (D) The Transwell assay shows the migrated glioma cells in different groups in U251 and LN229 cells. (E) Co‐culture Transwell assay shows the migrated macrophages in different groups in U251 and LN229 cells. (F) Statistical analysis of RT‐qPCR, EdU and Transwell assays in U251 and LN229 cells. *P < 0.05; **P < 0.01; ***P < 0.001; ****P < 0.0001.

An experimental validation was carried out due to the possible prognostic usefulness of SLFN12. The results of the RT‐qPCR assay demonstrated a considerable suppression of SLFN12 expression in the siRNA‐transfected groups of U251 (Figure 5A) and LN229 (Figure 5B) cells. The EdU assay demonstrated a significant decrease in proliferating glioma cells in the siRNA‐transfected groups of U251 and LN229 cells (Figure 5C,F). According to the Transwell assay, there was a significant decrease in migrating glioma cells in the siRNA‐transfected groups of U251 and LN229 cells (Figure 5D,F). Besides, the Transwell assay revealed significantly decreased migrating macrophages after coculture with tumour cells in the siRNA‐transfected groups in U251 and LN229 cells (Figure 5E,F).

### 3.6 Immune Features of SLFN12

**FIGURE 6:**
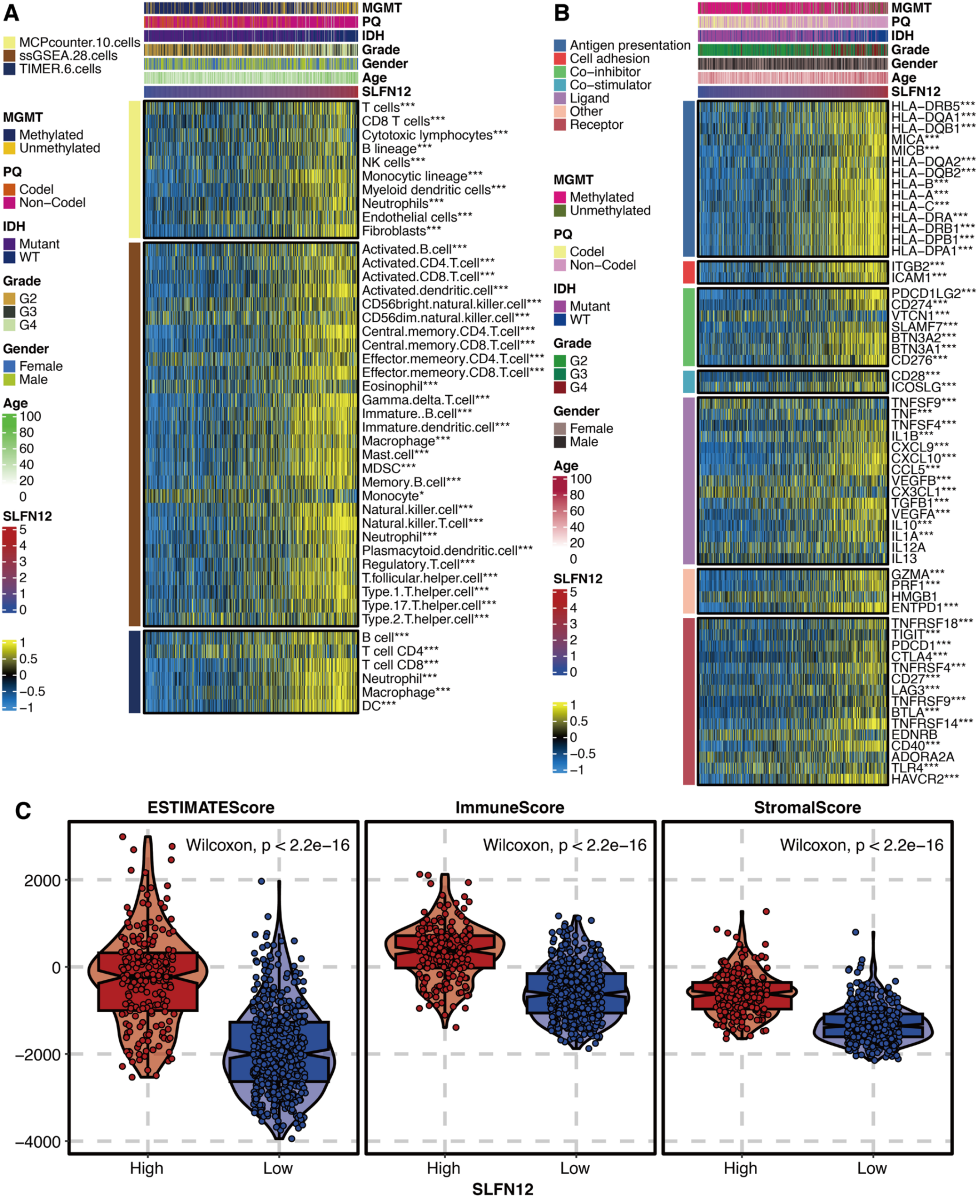
Immune features of SLFN12. (A) Correlation between SLFN12 and immune cells. (B) Correlation between SLFN12 and immune modulators. (C) Levels of microenvironment scores in high and low SLFN12 groups. *P < 0.05; **P < 0.01; ***P < 0.001; ****P < 0.0001.

Correlation between SLFN12 and immune cells revealed that SLFN12 was significantly associated with multiple immune cells, such as T cells, Myeloid‐Derived Suppressor Cells (MDSCs), mast cells, Regulatory T Cells (Tregs) and macrophages (Figure 6A). Correlation between SLFN12 and immune modulators revealed that SLFN12 was significantly associated with multiple immune modulators, such as ITGB2 and ICAM1 (Figure 6B). Besides, microenvironment scores were significantly higher in the high SLFN12 group (Figure 6C). Patients with elevated SLFN12 levels were associated with responses to anti‐PD‐1 immunotherapy, according to the Submap analysis (Figure S2).

### 3.7 Chemotherapy Drug Prediction of SLFN12

**FIGURE 7:**
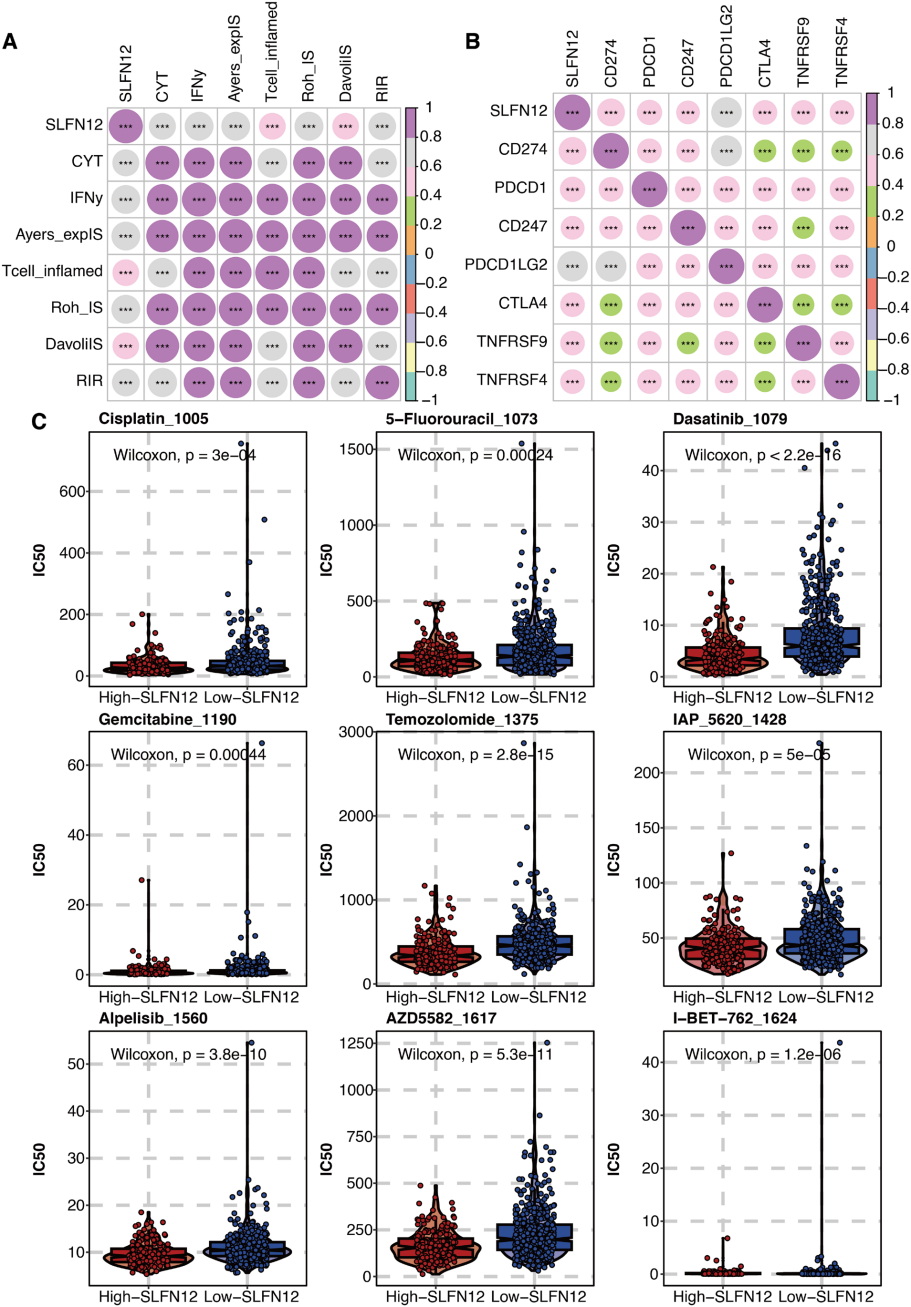
Chemotherapy drug prediction of SLFN12. (A) Correlation between SLFN12 and eight immune indexes. (B) Correlation between SLFN12 and classical immune modulators. (C) Estimated IC50 of chemotherapy drugs in high and low SLFN12 groups. *P < 0.05; **P < 0.01; ***P < 0.001; ****P < 0.0001.

SLFN12 was significantly associated with eight immune indexes, including CYT, IFNγIS, AyersExpIS, T cell–inflamed signature, RohIS, DavoliIS, RIR and ImmuneScore (Figure 7A). SLFN12 was significantly associated with classical immune modulators, such as CD274, CTLA4 and PDCD1 (Figure 7B). The estimated IC50 of chemotherapy drugs, such as Cisplatin, 5‐Fluorouracil, Dasatinib, Gemcitabine, Temozolomide, IAP, Alpelisib, AZD5582 and I‐BET‐762, was significantly higher in the high SLFN12 group (Figure 7C).

## 4 Discussion

This study employed a series of artificial intelligence‐related methods to identify a novel biomarker that predicts response to anti‐PD‐1 immunotherapy in glioma patients. Our findings demonstrate that expression of the SLFN12, as determined by our comprehensive analyses, is a strong and independent predictor of immunotherapy response in gliomas. Bioinformatics has been widely used for disease biomarker exploration [32, 33]. Further, machine learning has shown tremendous promise in disease research for various uses [6, 34]. Finding new biomarkers and prognostic signs has been a major focus [35, 36]. Our study on the immunotherapy response to gliomas is an example of how machine learning algorithms may analyse high‐dimensional information to find molecular markers that were previously unidentified but indicative of clinical outcomes. This is especially helpful for complicated, diverse cancer types because the data's natural noise and confounding variables frequently mask the underlying biological drivers of disease development and therapy response [37].

SLFN12 is a member of the Schlafen family of proteins, which have been implicated in various cellular processes, including cell growth, differentiation and immune regulation [38]. While the specific functions of SLFN12 are not fully elucidated, previous studies have suggested it may play a role in regulating T cell activation and proliferation [39]. Additionally, T cell activation was found to downregulate the expression of SLFN12 [40]. Our functional annotation analysis using Metascape further supports the association of SLFN12 with immune‐related pathways.

This study's prognostic value of SLFN12 expression in glioma is a key finding. We show that high SLFN12 expression significantly correlates with poorer overall survival in multiple independent glioma cohorts from two databases. The findings are generalisable across different populations and suggest that SLFN12 may serve as a novel prognostic biomarker for this disease. The finding that the estimated IC50 of several commonly used chemotherapy drugs, including cisplatin, 5‐fluorouracil, dasatinib, gemcitabine, temozolomide and others, was significantly higher in the high SLFN12 group is an important observation with potential clinical implications.

Glioma cells with high SLFN12 expression may be more resistant to various chemotherapeutic drugs, as indicated by the higher IC50 values showing decreased drug sensitivity. Numerous underlying processes, including increased drug efflux, altered drug metabolism, or deregulation of apoptotic pathways, could be blamed for this [41]. Given the importance of these chemotherapies in the standard of care for the management of gliomas, the reported drug resistance phenotype in the high SLFN12 group is especially noteworthy. For example, temozolomide is a key component of the Stupp protocol, the current first‐line treatment for glioblastoma that has just been discovered [42]. The lower overall survival seen in this patient subgroup may be related to the decreased susceptibility to temozolomide in high SLFN12‐expressing tumours. The SLFN12 biomarker may have clinical significance as evidenced by its decreased susceptibility to other chemotherapies, like gemcitabine [43] and cisplatin [44], which are occasionally included in salvage or combination regimens for gliomas. To overcome this chemoresistance, patients with high SLFN12‐expressing malignancies might need to consider different treatment approaches or closer monitoring.

Importantly, we also demonstrate that SLFN12 expression is a strong predictor of response to anti‐PD‐1 immunotherapy, with patients exhibiting high SLFN12 levels less likely to benefit from these treatments. The complex and heterogeneous nature of the tumour microenvironment in gliomas has made it challenging to identify robust, predictive biomarkers using conventional approaches [45]. The current study revealed the intricate relationship between SLFN12 expression and immunotherapy response, which may not have been discernible through traditional statistical analyses. These findings highlight the power of artificial intelligence techniques in uncovering novel insights from high‐dimensional cancer genomics data.

The mechanistic basis for the association between SLFN12 and immunotherapy response remains fully elucidated. One possibility is that SLFN12 may play a role in modulating the immune infiltration or functional status of the tumour microenvironment, thereby influencing the efficacy of immune checkpoint blockade. Our in vitro experiments demonstrating the impact of SLFN12 silencing on glioma cell proliferation, migration and macrophage recruitment provide preliminary evidence supporting this hypothesis. Previous studies have implicated that SLFN12 is involved in immune infiltration in gastric cancer [46]. Further investigation into these potential mechanisms will be essential for understanding the role of SLFN12 in macrophage activity and its implications for glioma immunotherapy responses.

## 5 Conclusion

In conclusion, our study has identified SLFN12 as a novel predictive biomarker for response to anti‐PD‐1 immunotherapy in glioma patients. These findings have important clinical implications, as they could inform patient selection and the development of more effective precision immuno‐oncology strategies for this devastating disease. Further mechanistic investigations and prospective clinical validation studies are warranted to fully elucidate the role of SLFN12 in glioma immunobiology and its potential as a therapeutic target. For instance, conducting multi‐centre studies to validate the prognostic and predictive value of SLFN12 in diverse patient populations will be crucial for establishing its clinical utility. Besides, investigating how SLFN12 expression changes throughout treatment and its correlation with treatment response and disease progression could provide insights into its role as a dynamic biomarker.

There are also limitations in our study. Our study used all glioma samples for analysis. Gliomas exhibit significant genetic and phenotypic diversity, which can impact treatment responses. Age, sex and ethnicity can affect immune responses and treatment efficacy. Differences in these demographics across cohorts may introduce biases that affect the generalisability of the findings. Besides, gliomas can be classified into different molecular subtypes (e.g. IDH‐mutant vs. IDH‐wildtype). These subtypes may respond differently to treatments, including immunotherapy, potentially confounding the association with SLFN12. Further study should focus on a specific type of glioma to enhance the generalisability of the findings.

## Author Contributions

**Zigui Chen:** data curation (equal), funding acquisition (equal), writing – original draft (equal), writing – review and editing (equal). **Chao Liu:** data curation (equal), writing – original draft (equal). **Wei Zheng:** data curation (equal), writing – original draft (equal). **Yuhua Fang:** methodology (equal), writing – original draft (equal). **He Zhang:** data curation (equal), writing – original draft (equal). **Jiawei Luo:** methodology (equal), writing – original draft (equal). **Jiale Li:** investigation (equal), writing – original draft (equal). **Yingqi Qiu:** software (equal), writing – original draft (equal). **Jun Peng:** investigation (equal), writing – original draft (equal). **Ying Xia:** funding acquisition (equal), supervision (equal), writing – review and editing (equal). **Changfeng Miao:** funding acquisition (equal), supervision (equal), writing – review and editing (equal). **Qisheng Luo:** funding acquisition (equal), supervision (equal), writing – review and editing (equal).

## Conflicts of Interest

The authors declare no conflicts of interest.

## Supporting information


Figure S1.

## Data Availability

The data that support the findings of this study are available from the corresponding author upon reasonable request.
