# Supplementary figures and images for: Deciphering the Role of SLFN12: A Novel Biomarker for Predicting Immunotherapy Outcomes in Glioma Patients Through Artificial Intelligence
Source: J Cell Mol Med. 2024 Dec 30;28(24):e70317. doi: 10.1111/jcmm.70317 (PMC11685066; doi:10.1111/jcmm.70317)

Supplementary Materials


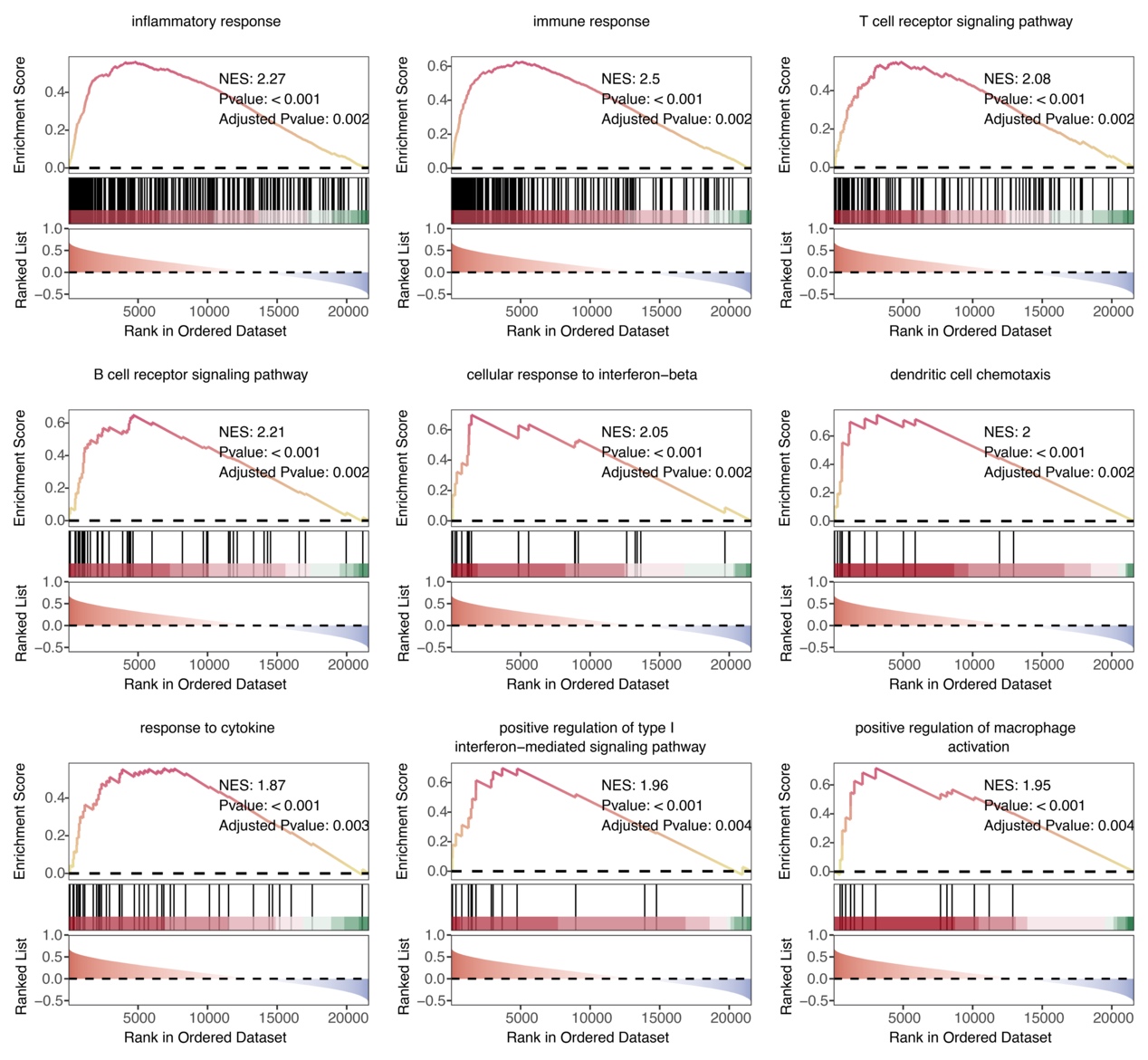


Figure S1. GSEA on SLFN12.


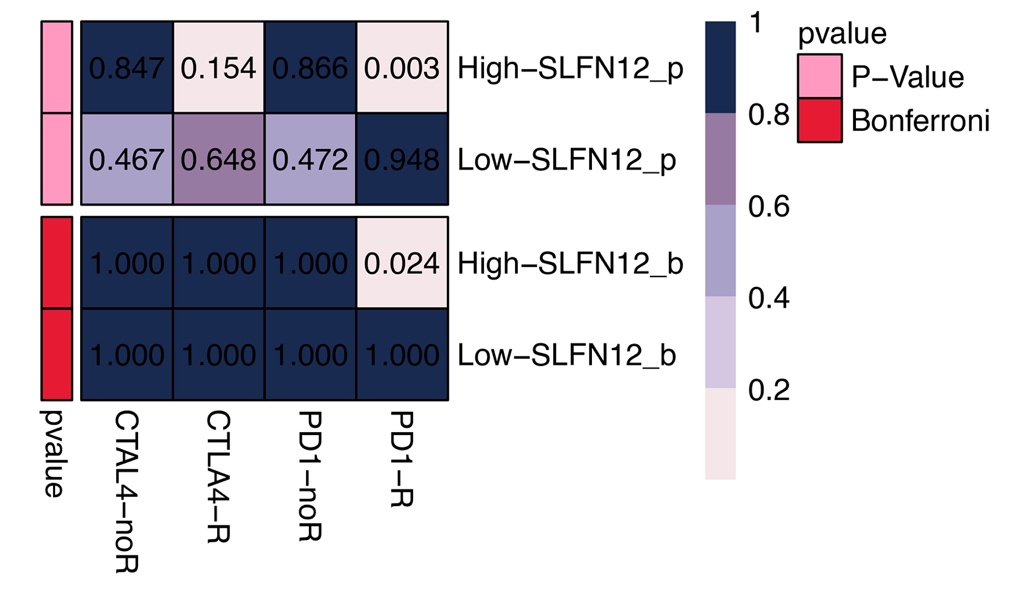


Figure S2. TIDE analysis on SLFN12.

Supplement: Supplementary file 1 — Figure S1. [file JCMM-28-e70317-s001.docx]
